# An Innovative Approach for Improving Information Exchange between Palliative Care Providers in Slovenian Primary Health—A Qualitative Analysis of Testing a New Tool

**DOI:** 10.3390/healthcare10020216

**Published:** 2022-01-22

**Authors:** Erika Zelko, Jozica Ramsak Pajk, Nevenka Krčevski Škvarč

**Affiliations:** 1 Institut for General Practice, Medical Faculty, University Linz, 4020 Linz, Austria; 2 Community Health Center Ljubljana, 1000 Ljubljana, Slovenia; jozica.ramsak-pajk@zd-lj.si; 3 Institute for Palliative Medicine and Care Maribor, Faculty of Medicine University Maribor, 2000 Maribor, Slovenia; nevenka.krcevski.skvarc@amis.net

**Keywords:** palliative care, family medicine, interprofessional collaboration, telemedicine

## Abstract

Background: Interprofessional collaboration is an important part of palliative care. Effective communication and information exchange is essential for a high quality of care. The aim of this study was to test the effectiveness of a new tool for exchanging information between professionals in palliative care on primary healthcare level. Methods: With suggestions from the experts regarding palliative care needs in an interprofessional team from the Delphi study and community nurses from the field, we developed a paper version of the tool. The paper version was tested in a pilot phase, and subsequently, we conducted ten semi-structured interviews with the users of the new tool to test its feasibility and usability. The data were analyzed using qualitative content analysis, leading to improvement and development of the digital version of the new tool. Results: After completing the pilot phase of the research, we identified the following four categories: a systematic tool for more consistent treatment and better communication during the patient’s visit; training and empowerment; quality, safety and digitalization; these categories were later included in the final version of the digital communication tool. Conclusion: Effective palliative care requires a good exchange of information and communication between all care professionals who work with the patient. Effective communication contributes to making patients and their relatives feel safe in their home environment and allows patients to stay in their homes even as their disease progresses. The systematical new tool was assessed as useful to improve interdisciplinary cooperation and prepared in a digital version. Further research after the long-term use of the developed digital tool in everyday work might confirm its sustaining importance.

## 1. Introduction

Despite the achievements of modern medicine, many diseases continue to evade a cure. Progressive chronic diseases, such as cancer, often cause disability, suffering and death. In total, 20,485 people died in Slovenia in 2018; of these, 17.2% (3527) died in nursing homes, 51.61% (10,573) died in medical institutions, 24.77% (5076) died at home and 6.4% (1309) died somewhere else. Thus, the present annual mortality rate of ~1.09% of the population is also expected to increase [1]. Our recently published study showed that 69.6% wish to die at home. According to the participants, patients at home need well controlled (93.8%) physical symptoms, medical and health care (65.9%), support from voluntary careers (41.4%) and 24-h consultation via telephone by a healthcare provider in case of problems (55.4%) [2]. 

Relief of suffering is the cardinal goal of palliative medicine and the patients’ most important expectation [3]. To improve the quality of life of patients and their families who face problems associated with life-threatening diseases, we must not only treat the pain but also assess and identify associated problems like physical, psychosocial and spiritual issues, in the early stages [4]. Studies show that palliative care in an early stage may slightly increase the quality of life of patients with advanced cancer. It may also decrease symptoms’ intensity to a smaller degree [5]. Palliative care requires health professionals from different disciplines to work together for the well-being of the patient and their family, using a collaborative model of care. Interprofessional collaboration involves paying attention to sharing, partnership and joint work [6]. Interdisciplinary collaboration has the capacity to affect both healthcare providers and patients. Research has shown that the lack of communication and collaboration may be responsible for nearly 70% of the adverse events currently reported [7]. Worldwide, there is a limited number of specialist palliative teams offering care to patients at the end of their lives, which means that many people receive palliative care from a primary care provider [8]. Providing care for end-of-life patients through primary care allows more people to die at home [9], and the continuity of care proved to reduce the number of acute medical visits at the end of life [10]. As shown by Pace et al., home care may represent an alternative to in-hospital care for the management of brain tumour patients, improve the quality of end-of-life care and reduce the costs of care [11]. Interdisciplinary collaboration and effective communication are two decisive elements determining the quality of community-based palliative care [12]. The usage of a structured communication method is associated with the improvement of information quality exchange [13]. In Slovenia the exchange of patient information is regulated by law [14] and not automatized or completely digitalized, even if the patient would agree with the information exchange. Hence, patients are responsible for the exchange of paper information between healthcare professionals. The need for effective information exchange is exceptionally high in emergency situations or when family physicians are not available to support a mobile palliative team or a community nurse. The COVID pandemic especially showed us the importance of an effective and uncomplicated exchange of information between healthcare professionals and interprofessional communication, with the aim to improve palliative and end-of-life care at the primary healthcare level. 

## 2. Materials and Methods

### 2.1. First Phase

Our research was divided into four phases. During the first phase, in 2018, we conducted a Delphi study. In total, 21 medical doctors, 13 women and eight men, participated in the study. The Delphi study was carried out to get a better understanding of the most important success and limiting factors in palliative care. In the first round they answered a couple of open questions regarding important information and data which are needed for quality and professional treatment of the patient. In the second round the verified topic related answers were sent to all participants. They were encouraged to rate the statements on a 5-point Likert scale. In the third round we assessed the statements that did not achieve the level of a 75% consensus (rating from 4 to 5). The remaining 20 statements were expected to be rated again by the participants on a 5-point Likert scale, but they also had their own rating and the median from the second round. The experts were chosen among the physicians who were included in palliative care at the primary healthcare level (emergency physicians, family physicians, palliative care physicians). The results were presented as a poster at a congress (Supplementary File S1). Based on the results of the Delphi study and in collaboration with a community nurse, we designed a paper version of the communication tool for people working with patients in palliative care (Supplementary File S2).

### 2.2. Second Phase

The paper version of the tool was pilot tested in the second phase of our research by the palliative teams caring for 20 patients. For participation in testing the paper version of the tool, we chose 5 teams.

Each team included a physician and a community nurse. They monitored four patients with the help of the paper version of the tool. One team worked in a nursing home, one in a rural family medicine practice and three teams were testing the tool in an urban region. The teams had basic knowledge about palliative care and were introduced to the paper version of the tool. We asked them also to make real-time notices about the field observation of the patients. After the pilot test, which lasted six months (three patients were included for six months; four patients were included for four months; seven patients were included for three months; five patients were included for two months and one patient was included for one month), we moved to the third phase of our research. 

### 2.3. Third Phase

We conducted semi-structured interviews with ten healthcare workers (five nurses and five physicians) who participated in the pilot testing and were willing to provide us with feedback about the usefulness and deficiencies of the tool. The participants were asked about their experience using the form at work, the shortcomings of the paper form and their improvement suggestions for the form. We used the following questions: How useful do you find the tool for everyday work? Did you identify any barriers to using the tool? Has the communication between palliative care providers using the tool changed? How was the experience with the relatives if you used the tool? Do you have any suggestions for adaptation of the tool for everyday use in the practice? We wanted to assess whether the use of the form affected the quality and safety of the treatment of patients who were included in the pilot testing of the form. We were interested in the impact of using the form on the exchange of information between the various members of the team treating the patient. We did not ask the patient and relatives directly about the usefulness of the form. We recorded most of the interviews and kept detailed notes on the two respondents who refused to be recorded. First, the data was coded and categorized. Usability testing was implemented based on a descriptive qualitative method [15] and content analysis, which aims to summarize the informational content of verbal and visual data [16,17]. This analysis was performed in a reflexive and interactive manner during the usability testing phase. The outcomes were included in the last (fourth) part of our research, where we developed the digital version of the tool. The observational notices from the fieldwork of the participants were also included in the development process of the digital tool version.

### 2.4. Fourth Phase

The tool was developed in cooperation with the computer company MediaInteractive Slovenia (Supplementary File S3 presents screen shots of the tool). The computer company took care of the safety of the environment and made it possible to integrate the program into the existing information system of the health institution. The program is designed to be used on a smartphone as well, which is a special advantage for use in the patient’s home. A more detailed description of the tool is beyond the scope of this article. The tool was accepted for use in the Public Health Centre Ljubljana and is waiting to be fully implemented in the daily work of the healthcare providers.

### 2.5. Ethical Concerns

Each participant received a code that was known only to the first researcher at the first coding process. The data were archived coded and so we ensured the anonymity of the participants.

## 3. Results

### 3.1. Delphi Study

The average age of the participants in the Delphi study was 44 years (from 31 to 67), and most of the participants worked in an urban area (70.0%). There were nine primary outcomes of the Delphi study (Table 1): 

### 3.2. The Pilot Phase–Testing the Paper Version of the Tool

The goal of the project’s pilot phase was implementing the tool in one nursing home, one rural family medicine practice and three urban family medicine practices.

## 4. Interviews after the Pilot Phase

After the tool had been used in practice for six months, we conducted ten semi-structured interviews with five family physicians and five nurses. The average age of the participants was 43 years; two were men and eight were women. The interviews lasted from 30 min to 1.5 h and were recorded, transcribed and analyzed thematically. All participants agreed to participate in the research. When analyzing the semi-structured interviews, we identified 37 codes, 13 themes and 4 categories (Table 2). 

### 4.1. Development of the Digital Tool

A total of 60% of the participants suggested the development of a digital model of the tool (Table 3). In cooperation with the computer company MediaInteractive, the digital model (Supplementary File S3) in ready for presentation and implementation. The company has already organized some presentations and a further one is possible after direct contact with the company (https://si.linkedin.com, accessed on 10 January 2022).

#### 4.1.1. Categories

##### A Systematic Tool for More Consistent Treatment and Better Communication during the Patient’s Visit

All interviewees (10/10) pointed out that the tool serves as a good reminder and introduces a systematic approach for obtaining key information during the patient’s visit. They also mentioned that it improves communication between the participants (8/10) and promotes more consistent tracking of the patient’s condition (6/10).

*“For me, continuous access to information is very important, it is good for the patients and the treatment…”*

*“Transparency of information, easier access to key information. There are benefits for home care of palliative patients, different physicians who treat the patient can quickly access the key information, such as therapies, action plan for complications, the patient’s wishes.”*

#### 4.1.2. Training and Empowerment

The respondents agreed (5/5) that every additional piece of information empowers the relatives who care for the patient. They stated that they had some problems filling out the form initially (4/10) and that they would need some additional palliative care training before continuing with a systematic use of the tool (8/10).

*“If the tool became widespread, training would be needed. I had some problems at the beginning.”*

*“This tool requires certain knowledge we unfortunately do not have. It would be good, considering that palliative care is a relatively new discipline and not very well researched in Slovenia, if the healthcare team received some training in this area.”*

#### 4.1.3. Quality and Safety

The participants mentioned how important it is that the patients and their relatives feel safe. The relatives felt calmer when the tool was used (5/10). The tool also served as a reminder for a systematic approach to patients in palliative treatment (7/10), which improved the quality of care [10,18,19,20].

*“Every additional piece of information available to the patients and their relatives increases the safety of everybody included and it decreases stress, if everything is available in one place, and makes it easier to respond constructively if any complications arise.”*

*“The tool helped the relatives know who to contact, who to ask. For example, a man felt nauseous, so his wife called me and, considering the medications they already had at home, I was able to talk to the physician by phone and resolve the problem immediately.”*

*“They welcomed the contact option, they felt more secure and they felt that the healthcare personnel really cares for them and the patient.”*

#### 4.1.4. Digitalisation

Many respondents (6/10) indicated that the tool should be introduced into the existing database and that we should prepare a digital version of the tool, which might be easier to use and more user friendly. Some (2/10) even suggested that we should include a separate section for the relatives to enter information about progressing conditions.

*“I think there should also be a section where a relative could enter the change in health condition.”*

*“We could have it in e-version, for example, in a cloud.”*

*“I would suggest that the tool becomes part of our IT system so that the entries are combined and immediately available to the physician. If something would have to be written down, the physician could handle it and then the nurse at the practice could communicate with me.“*

## 5. Discussion

In Slovenia, palliative medicine and care are in development. Palliative care is mostly provided by family physicians and community nurses. Community nurses and doctors often communicate only through a so called “work order document”. Such forms include a precise bullet point list of work packages from the physician, which a community health nurse has to conduct in order to provide stringent treatment progress, as physicians are difficult to access by phone in case of an emergency intervention. However, structured information sharing improves this interaction, as demonstrated by research abroad [12], and affects the effectiveness and quality of practice [18]. Because other services are often involved in providing treatment according to the patient’s needs, records and information are indirectly useful as well. Informal caregivers, hospices and spiritual support are also included in the provision of palliative care. In the Delphi study, the participants agreed that there is a need for a tool that would promote efficient information exchange during the patient’s visit and support better and safer care, allowing patients to stay at home if possible. The testing phase revealed that the users would prefer a digital version of the developed tool, which would extend the scope of telehealth services (video calls, monitoring of the parameters, communication with the patient and the relatives). Telehealth (the term is used interchangeably with telemedicine) is broadly defined as the use of telecommunication technologies to provide medical information and services [19]. Teleconsultation enhances communication between patients, families and palliative care teams, which reinforces their partnership, decreases the burden of the families and reduces the use of emergency services [20]. Several researchers have emphasized the importance of communication and interprofessional collaboration [21,22,23]. In our development process, we included emergency, family and palliative physicians into the first phase of the research and involved community nurses in the tool-design process. Unfortunately, in Slovenia, as well as elsewhere, the emergency department (ED) is often the first step into the healthcare system for patients with poorly managed symptoms of chronic diseases who are not currently in a hospital [24], meaning that EDs must frequently care for people in need of interventions that include palliative care and end-of-life care [25,26]. Emergency physicians in our research reported that a lack of information is a great problem when palliative care is advised. Accordingly, they have expressed a desire for better collaboration and information exchange between the professions, which would help them make some decisions on distance and reduce the number of unnecessary visits of palliative care patients to the ED. Similar findings were reported in another study, where end-of-life decisions were perceived as more complex in the absence of family or information about the patients’ end-of-life preferences or when there was a conflict with the relatives, time pressure or a lack of training in end-of-life decision making [27]. The participants in our study also stressed the importance of interprofessional collaboration and underlined the importance of defining the competences of all individuals involved in providing palliative care. They clearly stated which information they considered important for providing high-quality palliative and elderly care, like researchers in other studies with similar conclusions [21,22,23]. Efficient use of digital tools gives patients and their relatives better control over some disease symptoms [28,29,30,31]. The suggestion of our participants to allow patients and their relatives to use the tool is very appropriate. Australian researchers presented qualitative evidence demonstrating older people’s willingness to use mobile technologies to help them manage their pain; however, the needs for training in the use of the technologies and connectedness with clinicians were also highlighted [32]. In a study conducted in Great Britain, the participants did not understand why out-of-hours providers could not access further information about their medical histories, given the level of computerization within the National Health Service [33]. Considering such information, our study is crucial, as it focuses on improving information exchange between different professional groups involved in palliative care. Lack of medical professionals is a huge problem in most European countries and very often the informal caregivers are the ones to care for chronically ill and palliative patients. Our model could help them to reach medical professionals easier and more effectively. The concept and terminologies of the model are easily understandable, so it could also be used in this population of potential users of the tool.

### Limitation of the Study

One of the important limitations of our study was the small number of included participants and a short period of testing the paper version of the tool. Among Slovenian healthcare providers, there are more women than man, which is also visible in our sample. We could not exclude this part as a bias of our work. We didn’t test the developed digital version of the tool because the pandemic has stopped the implementation. Some of the participants addressed the need of introducing the tool in more depth before widespread use. The possibility to include open comments from in the model could ensure greater visibility of the needs of patients in palliative care as well as the needs of their relatives. The cost-benefit aspects of implementing the model should be considered in further studies, especially regarding unnecessary hospitalizations, home deaths and costs of the equipment for health professionals and patients. 

## 6. Conclusions

According to predictions based on demographic data, more than one-third of the Slovenian population will be older than 65 years of age in 2057 [34]. Hence, we can expect an increase in chronic patients and those needing palliative care. Efficient palliative care requires good communication between all experts who care for the patient, which makes patients and their relatives feel safe in their home environment and allows patients to stay at home despite the progression of their disease. Our tool proved useful for improving interdisciplinary collaboration between individual providers for patients receiving palliative care. The limitations of our project include the short period of use, the low number of participants and the lack of practice in the use of digital tools. However, improvement is always possible. As such, we will continue to monitor the usability of our tool in practice. 

## Figures and Tables

**Table 1:** Primary outcomes of the Delphi study.

Number	Primary Outcomes of the Deplhy Study
1	Patients’ personal data, including their preferences and social background
2	Professional support, including the names and contacts of important professional care providers
3	Documents, including the hospital dispatch letter, personal ambulance card and completed questionnaires
4	Disease section, including the patients’ background, current symptoms, therapy and quality of life
5	Therapeutic treatment plan, including the current course of care, treatment and palliative plan
6	Information exchange section, including important up-to-date information
7	Section on examination, providing data about current and planned medical examinations
8	Indicators of the quality of care, where all examinations, hospitalizations, urgent calls and treatment regimen would be noted
9	Cooperation section, which would promote mutual respect and trust, good information flow, professionalism and clearly defined tasks and objectives for every team member

**Table 2:** Results of the analysis of the semi-structured interviews, which were conducted after pilot testing of the paper version of our communication tool.

Codes	Themes	Categories
Consistent tracking of patient’s condition Systematic approach Reminder Easier to estimate the condition More efficient resolution of complications All important information in one place (contacts) Need for a specific tool for nursing homes Larger range of services and medical aids A connecting tool More efficient exchange of information	Reminder Systematic approach Continuity Specific tool Efficient communication	A systematic tool for more consistent treatment and better communication during the patient’s visit
Additional personnel training needed Unfamiliar rating forms Complex rating scales Time consuming when used for the first time, then it gets better Empowerment of co-workers and relatives Simpler communication once the family and the relatives adopt the form It would help if the doctor knew the tool It helps considerably if the relatives are familiar with palliative care and the tool Understanding Transparency Time consuming	Personnel training Collaboration across disciplines and professions Empowerment of co-workers and relatives	Training and empowerment
Improved quality of care Better communication between stakeholders—interprofessional collaboration Better collaboration with the family Transparency of treatment and important information Relatives feel more secure Fewer explanations over the phone Relatives feel supported by the healthcare workers and have more access to them. Allows the relatives and the patients to express their wishes (nursing home, family meeting) Access to important people Fewer activations of emergency service (EMS)	Treatment quality and safety Safety of the patient and the co-workers Relatives’ trust Better access to healthcare personnel in critical moments	Quality and safety
Digitalisation The tool needs to be flexible and adaptable Connection to the existing IT system Applicable Time for implementation Larger table for therapy in the digital format	Digitalisation	Digitalisation

**Table 3:** Summary of the opinions from interviewees after pilot study.

Opinions (*n* = 10)	Percentage/%
Tool serves as good reminder for information collection	100
Tool enables better communication	80
Better tracking of the patients’ condition	60
Empowerment of relatives	100
Some problems with fulfilling the form	40
Recognized need for additional palliative care training	80
The tool should be introduced into the existing database	60
Digital tool might be easier to use	60
The separate section should be introduced for the relatives’ reports	20

## Data Availability

The data presented in this study are available on request from the corresponding author. The data are not publicly available due to [language of it].

## Supplementary Materials

The following supporting information can be downloaded at: https://www.mdpi.com/article/10.3390/healthcare10020216/s1, File S1: Conference Poster-Delphi study File S2: Paper Version of the tool; File S3: Screenshot of the digital tool version.
