# Supplementary material for: An Innovative Approach for Improving Information Exchange between Palliative Care Providers in Slovenian Primary Health—A Qualitative Analysis of Testing a New Tool
Source: Healthcare (Basel). 2022 Jan 22;10(2):216. doi: 10.3390/healthcare10020216 (PMC8872608; doi:10.3390/healthcare10020216)
Supplement: Supplementary file 1 [file healthcare-10-00216-s001.zip › healthcare-1530663-supplementary File S1.pdf]

Supplementary File S1

Topic: Paliative care
Presentation: Poster

DELPHI STUDY ABOUT PALIATIVE CARE

PhD Irena Makivić, Erika Zelko, Jožica Ramšak Pajk

Introduction/Background

The cooperation between primary, secondary, or tertiary health care facilitators is important, to manage quality palliative patients' care. Palliative does not refer only to terminal phase of the care, but also home-based management of chronic diseases.

Objective/Methods

The Delphi study was carried out to get the picture about important factors in palliative care. In the Delphi study 21 medical doctors cooperated, of whom 13 were women and 8 were man. In the first round they answered on some open questions about important information and data that are needed for quality and professional treatment of the patient; about the important questionnaires and documents that are needed in good palliative care. In the second round all grouped answers were sent to all participants and they could answer on a 5-point Likert scale. In the third round there were the statements that did not achieve the level of 75 % consensus on the agreement (rating from 4 to 5). On those 20 statements the participants were expected to answer again on a 5-point Likert scale, but they also had the median from the second round and their own rating.

Results

Average age of the participants was 44 years old (from 31 to 67) and they mostly worked in an urban area (70.0 %). The outcome of the study were nine big areas: (1) Patients' personal data about their wishes and social background; (2) Professional support with the names and contacts of the important professionals; (3) Documents with Hospital dispatch letter, personal ambulance card and completed questionnaires; (4) Disease field with the patients' background, current symptoms, therapy and patients' quality of life; (5) Therapeutic treatment plan with the current course of care, treatment and palliative plan; (6) Information part which is including important up-to-date information; (7) Investigation field about current and planned medical examinations; (8) Indicators of quality care where are all examinations, hospitalisations, urgent calls and treatment regimen subscribed; and the last one was the (8) Cooperation which is including mutual respect and trust, good information flow, professionalism and clearly defined tasks and objectives from the team members.

Conclusion

With the help of the Delphi study, we managed to develop the important fields for patient's management at home.
