# Supplementary material for: An Innovative Approach for Improving Information Exchange between Palliative Care Providers in Slovenian Primary Health—A Qualitative Analysis of Testing a New Tool
Source: Healthcare (Basel). 2022 Jan 22;10(2):216. doi: 10.3390/healthcare10020216 (PMC8872608; doi:10.3390/healthcare10020216)
Supplement: Supplementary file 1 [file healthcare-10-00216-s001.zip › healthcare-1530663-supplementary File S2.pdf]

Supplementary File S2 : Plan of palliative care for patients at home

Patients' data

Name: ______________________________
Insurance number: ______________________________
Address: ______________________________
Birthday: ______________________________

Family members/ caregivers:

Name	Relationship	Contact

Important contacts

Personal Family physician	________	Tel.:	________	HC	________
Community nurse	________	Tel.:	________	HC	________
Palliative physician	________	Tel.:	________	HC	________
Leading Clinicians	________	Tel.:	________	HC	________
Social Worker	________	Tel.:	________	Social Centre	________

Important medical Data

Birthday	Diagnosis	Last hospitalization	Informed with the disease status (Yes/No)

All currently diagnoses

____
____
____
____

Medication

Oxygen: ________________________________

Medication	Doses	application	purpose

Rescue doses in case of complications

Symptoms	Medication	Doses	Application art/ Suggestions/agreement
Pain			
restlessness			
Insomnia			
Shortness of breath			
nausea			
Weakness			

Diet ____

Food supplements ____

Allergy ____

Family conference (Nurse, physicians, patient, relatives)

planned/performed/not planned: ____

Date: ____ ________________

Short summary of the family conference:
(for example – plan of care, what to do in case of complications, hospitalization, Hospice, patients' needs and wishes, preferred place for dying)

____
____
____

Patient’s wishes

Should the relatives be informed about the situation?

☐ no ☐ yes

The relatives take part at care:

☐ no ☐ They are actively involved ☐ They need help ☐ They need more knowledge ☐ They are not able to take part in care

He relatives need psychosocial support

☐ no ☐ yes

Has the patient filed out the advance care planning formular?

☐ no ☐ yes

If yes, please add the date and Hour: ___________________________________

Is patient aware about the prognosis?

☐ no ☐ yes

Please choose the number that best describe your currently feeling.

Without pain	0	1	2	3	4	5	6	7	8	9	10	The worst possible pain
Without fatigue<br>(fatigue = loss of energy)	0	1	2	3	4	5	6	7	8	9	10	The worst fatigue possible
Without insomnia	0	1	2	3	4	5	6	7	8	9	10	The worst possible insomnia
Without nausea	0	1	2	3	4	5	6	7	8	9	10	The worst possible nausea
Without loss of appetite	0	1	2	3	4	5	6	7	8	9	10	Completely loss of appetite
Without dyspnoea<br>(dyspnoea= shortness of breath)	0	1	2	3	4	5	6	7	8	9	10	The worst dyspnoea
Without depression<br>(depression = sense of hopelessness)	0	1	2	3	4	5	6	7	8	9	10	High depression
Without anxiety<br>(anxiety = sense of nervousness)	0	1	2	3	4	5	6	7	8	9	10	High nervousness
High well-being	0	1	2	3	4	5	6	7	8	9	10	The lowest well-being
Without __________<br>(Other symptoms like obstipation)	0	1	2	3	4	5	6	7	8	9	10	Very high_______________

Who has filed out the questionnaire?

☐ patient
☐ caregiver (relatives, family)
☐ health professional (physician, nurse)
☐ patient with help of the caregivers

Kanefsky score

Which medical equipment use the patient?

Breathing	Oxygen
	Trachea- stoma
Digestive tract	Gastrostomy
	Ileostomy
	PEG
	Nasogastric stoma
Excretion	Urostomy
	Nephrostomy
	Incontinence
	Katter
Skin	Ulcus
	Decubitus
	Periferny canal
	Elastomer pup
	Porths (PIC)

Date ____________________ ***Coordinator*** ____________________

For the physician or nurse to be filed out

Currently status

Date	Patients' feelings/status		Interventions (phone consultations, interventions, change of medication and care goals)
	po Edmonton	Kanefsky	

Date	Medical care problems	Interventions / measures	Assessment and redefinition of the care plan and goals

To be filed out from the nurse

Home visits of other healthcare professionals

Health profession	Intervention	Date

The formular prepared: mag. Jožica Ramšak Pajk, doc. dr. Erika Zelko, dr. Irena Makivić
