# Supplementary material for: An Innovative Approach for Improving Information Exchange between Palliative Care Providers in Slovenian Primary Health—A Qualitative Analysis of Testing a New Tool
Source: Healthcare (Basel). 2022 Jan 22;10(2):216. doi: 10.3390/healthcare10020216 (PMC8872608; doi:10.3390/healthcare10020216)

Supplementary File S3

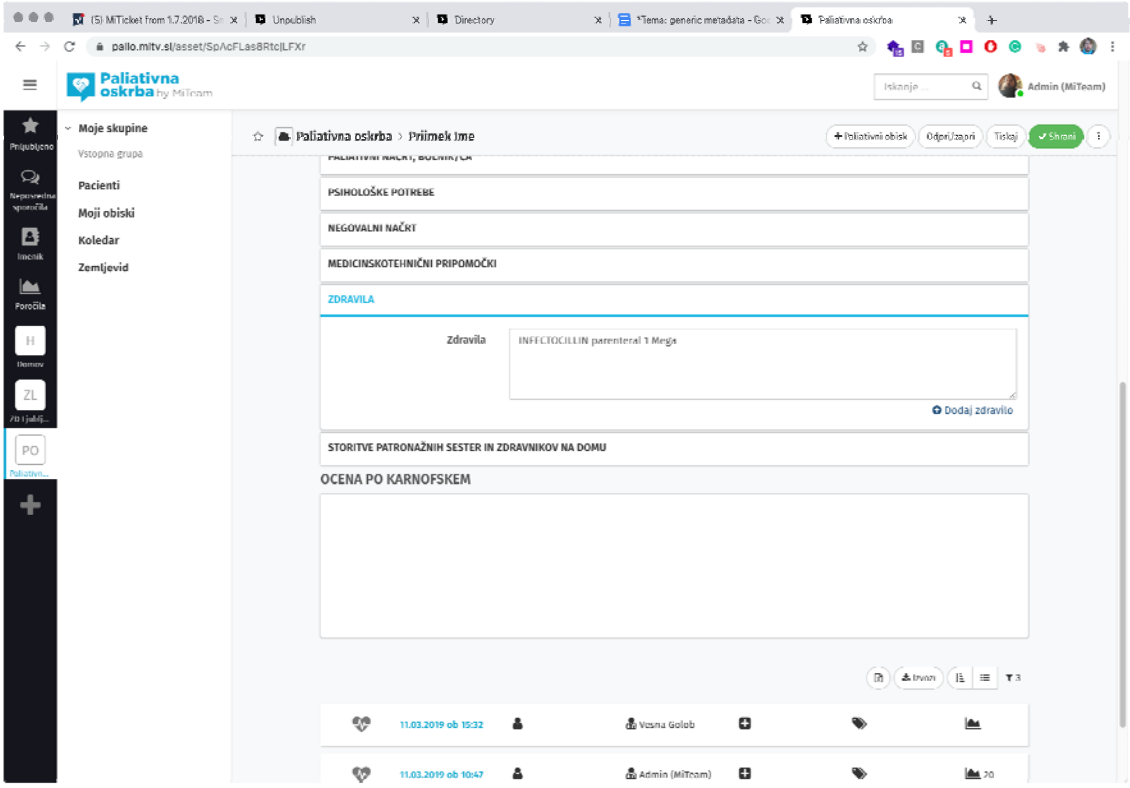

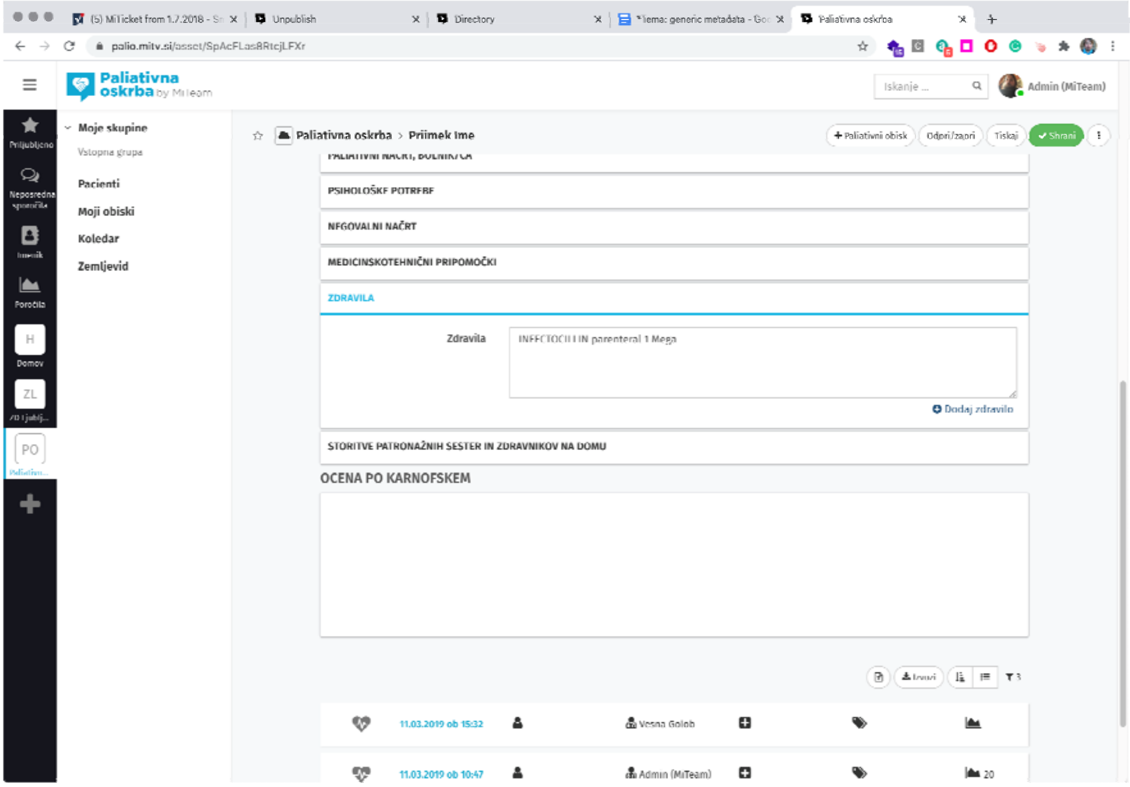

Supplement: Supplementary file 1 [file healthcare-10-00216-s001.zip › healthcare-1530663-supplementary File S3.pdf]
